# Salmonella and Escherichia coli contamination of poultry meat from a processing plant and retail markets in Ibadan, Oyo State, Nigeria

**DOI:** 10.1186/2193-1801-3-139

**Published:** 2014-03-12

**Authors:** Gladys Taiwo Adeyanju, Olayinka Ishola

**Affiliations:** Department of Veterinary Public Health and Preventive Medicine, University of Ibadan, Ibadan, Nigeria

**Keywords:** Poultry meat, *Salmonella* spp, *Escherichia coli*, Antibiotics, Resistance

## Abstract

*Salmonella* spp and *Escherichia coli* are the two most important food-borne pathogens of public health interest incriminated in poultry meat worldwide. This study is to access their levels in frozen poultry meat obtained in Ibadan, Oyo State and compare those obtained from a commercial Nigerian-registered poultry company having a broiler-processing plant, Sayed Farms Ltd(R), with that obtained from retail stores. These retail stores source their products as illegal imports from neighboring Benin Republic or Togo because of a ban imposed by Government policy in Nigeria since July 2002 (USDA, GAIN report #NI2025:1-6, 2002).

Microbiological Standards and Guidelines by USDA (National Agricultural library) (USDA 2011) and NCCLS guidelines (from Global Salm-Surv, 2003) were used during the research work. The study was approved by the Ethical Research Review Board (ERRB, Research Management Office 2011), University of Ibadan, Nigeria. A total of one hundred and fifty-two (152) frozen poultry meat samples comprising ninety-nine retail poultry (53 chicken and 46 turkey) and 53 chicken from the processing plant were accessed. ISO Standards catalogue 07.100.30 (2011) was used in accessing the levels of *Salmonella*, *Escherichia coli*, Enterobacteriaceae counts and Aerobic plate count. ISO 6579: 2002 was used for *Salmonella* isolation and ISO-16654:2001 for *Escherichia coli* isolation.

There was a higher level of Aerobic plate counts and Enterobacteriaceae counts in frozen retail poultry meat than from the processing plant. *Salmonella* contamination from the ninety-nine poultry samples (53 chicken and 46 turkey) obtained from retail markets was at 33% [chicken 32.1% (17/53) and turkey 34.8% (16/46)] while *Escherichia coli* at 43.4% [chicken 47.2% (25/53) and turkey 39.1% (18/46)]. From the processing plant, twelve (12) *Salmonella* isolates were obtained and prevalence rate calculated as 22.6% while three (3) *Escherichia coli* isolates at 5.7% was obtained.

Antibiotic sensitivity for isolates using eight different Gram-negative antibiotics showed different resistance patterns. Nitrofurantion and augmentin showed a decrease in their sensitivity to isolates than they normally should. *Salmonella enterica* spp. showed 93% resistance to tetracycline and 100% resistance to augmentin and amoxicillin, while *Escherichia coli* showed 100% resistance to augmentin and amoxicillin.

## Introduction

Turkey, spent layers and majorly, broilers serve as sources of poultry meat which has become a product accepted and consumed worldwide as there is an absence of cultural or religious obstacles associated with their use as food and Nigeria is no exception (Alabi and Alabi 2009).

Major exports of poultry to West Africa comes from the European Union (EU), the exports of which has increased from 12500 tons in 1996 to 86000 tons in 2003, mainly to Benin, followed by Ghana, Nigeria, Senegal, Togo and Ivory Coast. Imports into Nigeria has been banned since July 2002 in order to boost local production, but it continues although undocumented (USDA, GAIN report 2002). The European community (EC) was banned from the use of antimicrobials as growth promoters in poultry faming in January 2006 because of the risks of increased resistance (Miranda et al. 2008) but demand and imports of poultry meat into Nigeria has increased over the years. Nigeria annually produces an average 11829 tons of chicken, spending about 8 million US dollars to import chicken meat in 2005 alone (Alabi and Alabi 2009). Poultry meat (chicken and turkey) is readily available for sale in retail markets areas such as is obtainable in Ibadan, Oyo state, Nigeria. These imported poultry are kept in freezers but power supply is not constant and is augmented by the use of fuel-powered generating sets. It should be noted however that ensuring consumer health concerns the greater involvement of the health sector, development of Codex standards, guidelines and incorporation of the work of the Commission into the national legislation to promote food safety and fair trading practices as should be reflected in the priorities of the Codex Alimentarius Commission of any country especially in the developing countries.

During the slaughter of poultry birds, there can be fecal contamination of the carcasses from the gut of these birds which means bacteria present in the spilled gut content is passed on as contaminants. Of importance is the coliforms especially *Escherichia coli* and *Salmonella*. Collibacillosis and *Salmonellosis* have been described as the leading causes of food-borne illnesses worldwide (Panisello et al. 2000), therefore, it becomes important that ensuring consumer health concerns the greater involvement of the health sector.

*Salmonella* is of an increasing public health concern because they are the most incriminated pathogenic microorganisms of bacterial food poisoning especially present in poultry meat, with infection being through the handling of raw poultry carcasses and products, together with the consumption of undercooked poultry meat (Panisello et al. 2000).

The modernization of chicken farms and globalization of the bird breeding trade also have played a role in infection (Velge et al. 2005), with several serotypes being isolated from retail poultry products from many years back in various parts of the world (Rindhe et al. 2008). Prevalence of *Salmonella* in poultry meat using both traditional and conventional methods has been reported worldwide from retail outlets, retail markets and processing plants. It has been reported to be as low as 1.56% from a Morocco poultry processing plant (Cohen et al. 2007) and as high as 20% from a poultry processing plant in USA (Russell 2009). In retail markets, prevalence was reported in broilers at 10.60% in Croatian market (Kozačinski et al. 2006), 31% in India (Dahal 2007), 35.5% in Mexico (Miranda et al. 2009) and 5.92% in Saudi Arabia (Moussa et al. 2010). In Nigeria, several rates have been reported with 11.1% prevalence in Calabar metropolis (Ukut et al. 2010) and 2% in Osogbo (Adesiji et al. 2011). Resistance to ampicillin appears to be the most common in Nigeria, followed by trimethoprim-sulphamethozazole, streptomycin, cephalexin, gentamycin (Enabulele et al. 2010) and more than 90% resistance to tetracycline (Sakaridis et al. 2011).

*E. coli,* a natural inhabitant of the intestinal tracts of humans and warm-blooded animals, is used as an indicator bacterium because it acquires antimicrobial resistance faster than other conventional bacteria (Miranda et al. 2008). Its presence therefore reliably reflects faecal contamination, indicating a possible contamination by enteric pathogens. Many different types of foods are sources of the bacterium and have been identified as a potential source of Shiga Toxin-producing *Escherichia coli* (STEC) for which such raw or undercooked foodstuffs get contaminated either during primary production (e.g. slaughtering) or further processing and handling (e.g. cross contamination during processing, human-to-food contamination via food handlers). *E. coli* has been isolated worldwide from poultry meat (Canton et al. 2008; Adesiji et al. 2011), probably due to the increased usage of antimicrobials (Miranda et al. 2008). Percentage prevalence in poultry meat has been variable depending on method and media used in its isolation. 19% prevalence was observed in South Africa (Dahal 2007), 48.4% in Morocco (Cohen et al. 2007) and as high as 98% in India (Saikia and Joshi 2010). In Nigeria, 16% has been isolated in Osogbo (Adesiji et al. 2011) and 11.1% in Calabar metropolis (Ukut et al. 2010). Enteroaggregative *E. coli* (EAEC) and Enterotoxigenic Fluoroquinolone resistant *E. coli* strains often show resistance to other drugs such as ampicilin, tetracycline, chloramphenicol, trimethoprin, sulphamethoxazole and Gentamicin (Komp et al. 2003), with a significant increase in fluoroquinolones resistant *E. coli* in many countries over the last few decades (Viroy et al. 2005). In Nigeria, the same results of *E. coli* resistance to quinolones and the other drugs have also been identified from poultry and beef sources (Olatoye 2010; Adesiji et al. 2011; Adetunji and Isola 2011). It has therefore been suggested that the resistance to the penicillin group of drugs, tetracycline, chloramphenicol and gentamicin may have been precipitated by the resistance to the quinolones (Komp et al. 2003).

## Materials and methods

### Sample collection

The study was carried out in Ibadan metropolis comprising Ibadan North, Ibadan North-West, Ibadan North-East, Ibadan South-West and Ibadan South-East. Sayed Farm Ltd®, is a commercial poultry company located in Ibadan South-East, registered in Nigeria, having a hatchery and a processing plant. The hatchery is involved in the hatching of both meat birds (broilers) and egg-layer birds, while the processing plant is involved in processing table-size broilers which are frozen before been sold.

A total of one-hundred and six chicken were sampled (fifty-three samples from retail markets and fifty-three samples from the processing plant) using Thrusfield method (Thrusfield 2005) while forty-six turkey were randomly sampled from the retail market. Research work was carried out within the months of April to July 2011.

Samples obtained were placed in sample-collection bags, properly labeled, stored in a cold box and taken to the laboratory for immediate analysis. However, no reference strains were used as quality control during the microbiological detection and isolation. One-way ANOVA and multiple comparisons were the statistical tools used while disk diffusion method was used for antibiotic sensitivity.

Microbiological Standards and Guidelines by USDA, (National Agricultural library) (USDA 2011) and NCCLS guidelines (from Global Salm-Surv, 2003) were used during the research work. The study was approved by the Ethical Research Review Board (ERRB), University of Ibadan, Nigeria (ERRB, 2011).

### Microbiological procedure

#### Aerobic plate count (APC) and Enterobacteriaceae counts (ENT)

25 g of test sample (meat) was weighed and blended in a stomacher machine for 2 minutes. A gram of the sample was weighed out and homogenised in 9mls buffered peptone water (LabM, UK) to give a dilution of 1:10. A six-fold serial dilution was then prepared. 0.1 ml of dilutions 10^-6^ and 10^-5^ for every sample was respectively plated on plate count agar (PCA) (Biomark, India) for aerobic plate count determination and on McConkey agar (MCA) (LabM, UK) for Enterobacteriaceae (enteric bacteria) counts. The PCA and MCA were both incubated overnight (18–24 hours) at 37°C.

Distinct colonies on PCA and MCA were counted using a digital colony counting chamber and recorded in colony forming units per gram (cfu/g) of meat sampled using the formula:


These were further expressed in mean colony forming units per gram (mean cfu/g) and converted to log 10 base values. Statistically, SPSS statistical tool using One-way ANOVA and multiple comparisons were calculated.

#### *Salmonella*detection and isolation

ISO-6579: 2002 food microbiology procedure employing the horizontal method for the detection of *Salmonella* from food and animal feeding stuffs was used (ISO Standards catalogue 07.100.30; WHO 2010).

- Non-selective pre-enrichment: 25 g of meat sample was collected under sterile conditions and transported to the laboratory in a maintained cold chain. It was blended in a stomacher machine for 2 minutes and a gram of the sample was weighed out and homogenised in 9 mls buffered peptone water (LabM, UK) in a test-tube to give a dilution of 1:10. Test-tubes were corked properly, labelled and incubated overnight (18–20 hours) at 37°C.
- Selective enrichment: Rappaport-Vassiliadis soya peptone (RVS) (Oxoid, England) was prepared. On cooling, 10mls was dispensed into test-tubes. 0.1 ml of the pre-enrichment was taken using a sterile pipette and transferred into the test-tube containing the Rappaport-Vassiliadis soya peptone broth. Test-tubes were covered using a cork, labelled and incubated overnight (18–24 hours) at 41.5°C ± 0.5°C.
- Selective agar plating: A 10 μl wire loop was used to pick a loop-full volume from the RVS broth and inoculated unto already prepared and solidified Brilliant green agar (BGA) (LabM, UK). Plates were spread out, labelled, inverted and incubated at 37°C overnight (18–24 hours).
- Sub-cultivation: suspect colonies which caused the colour of the medium to change from yellow to red/pink were sub-cultivated unto nutrient agar (LabM, UK) and incubated overnight (18–24 hours) at 37°C.

Serotyping was carried out to determine incriminated specie for *Salmonella* using Polyvalent O Antisera. Two seperate loopful of normal saline was placed on a clean glass slide after which a clean wire loop was used to take a small part of suspect colony grown overnight on nutrient agar and placed on both drops of the normal saline and thoroughly mixed. A loopful of the antisera was placed on one of the bacteria suspension while normal saline was placed on the other (control). Both bacteria suspensions were mixed using a sterile wire loop and gently tilted to observe for agglutination. Positive *Salmonella* spp. with somatic antigen is indicative of agglutination reaction.

#### *Escherichia coli*isolation

ISO-16654:2001 food microbiology procedure of the horizontal method for the detection of *Escherichia coli* O157 from food and animal feeding stuffs was employed in this study (ISO Standards catalogue 07.100.30; WHO 2010).

- Enrichment: 25 g of meat sample was collected under sterile conditions and transported to the laboratory in a maintained cold chain. It was blended in a stomacher machine for 2 minutes and a gram of the sample was weighed out and homogenised in 9mls buffered peptone water (LabM, UK) in a test-tube to give a dilution of 1:10. Serial six-fold dilution in peptone water was prepared (10^-1^ to 10^-6^) for test samples. 0.1 ml of dilutions 10^-6^ and 10^-5^ for every sample was respectively plated on the MCA and spread out using glass spreader. Plates were labelled, inverted and incubated overnight (18–24 hours) at 37°C.
- Sub-cultivation: Presumptive pinkish colonies (coliform) obtained from incubated plates were sub-cultivated unto the fresh MCA plates. Plates were labelled, inverted and incubated overnight (18–24 hours) at 37°C.
- Plating on Nutrient agar: Isolates were transferred into nutrient slants, labelled and incubated overnight (18-24 hours) at 37°C. On removal from the incubator, slants were stored in a refrigerator at 4°C, ready for biochemical analysis.

#### Biochemical tests

Catalase test, sugar fermentation using TSI (LabM, Uk), Mannitol test and Gram staining were carried out for *Salmonella* spp. while catalase test, sugar fermentation using TSI (LabM, Uk), Kovac’s test and Gram staining were employed for *Escherichia coli* isolates.

#### Antibiotic sensitivity test for isolates

Disk diffusion antibiotic sensitivity test was used in determining sensitivity of the *Salmonella* and *Escherichia coli* Gram-negative bacteria isolates obtained to various antibiotics at different micrograms. Augmentin (30 μg), Ofloxacin (5 μg), Gentamicin (10 μg), Nalidixic acid (30 μg), Nitrofurantoin (200 μg), Cotrimaxazole (25 μg) and Tetracycline (25 μg) were used to determine sensitivity pattern.

## Results

### Aerobic plate count and Enterobacteriaceae counts of frozen poultry

#### Aerobic plate counts and Enterobacteriaceae counts in chicken

Mean (cfu/g) ± SD aerobic plate count (APC) for all the LGA’s was calculated as 7.497 ± 0.94. However, there was no significant difference when one local government was compared with another using ANOVA. The only significant difference noted using LSD was between Ibadan South-East and Ibadan North-west at p value of 0.05 and between Ibadan South-West and Ibadan South-East at p value of 0.006 using multiple comparisons. Using ANOVA and multiple comparisons, no significant differences in ENT counts was observed when the means were calculated individually for each LGA and when they were compared one to another. Mean (cfu/g) ± SD Enterobacteriaceae counts for all the LGA’s was calculated as 6.76 ± 1.93. Table 1 also shows logarithm mean counts in cfu/g.

**Table 1:** **Aerobic plate counts and enterobacteriaceae counts for chicken meat in Ibadan metropolis**

Ibadan LGA	Sample size	Aerobic plate count cfu/g	Enterobacteriaceae count cfu/g
	n	Mean ± SD	Mean ± SD
Ibadan North	16	7.45 ± 0.48	6.63 ± 2.42
Ibadan North-West	5	7.80 ± 0.37	6.55 ± 1.76
Ibadan North-East	8	7.57 ± 0.42	5.97 ± 2.78
Ibadan South- West	14	7.88 ± 0.61	7.33 ± 1.08
Ibadan South-East	10	6.81 ± 1.79	6.90 ± 1.26
Total	53	7.49 ± 0.94	6.76 ± 1.93

key: LGA = Local Government Area, n = sample numbers collected, SD = standard deviation, cfu = colony forming unit, g = gram.

**Figure 1:**
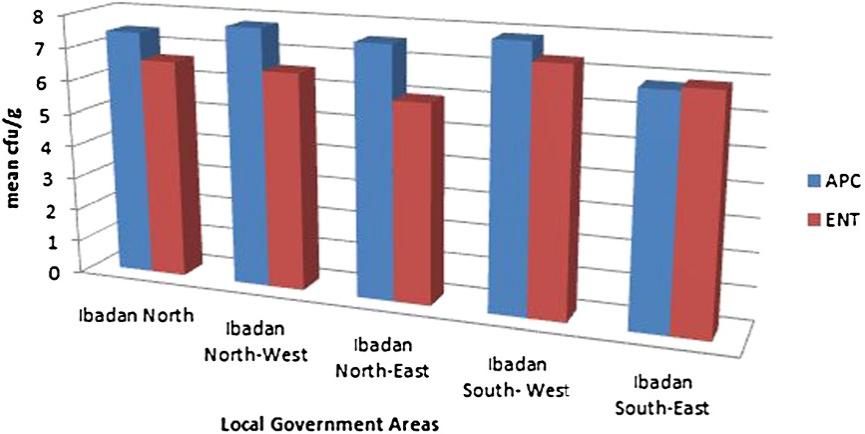
**Aerobic plate counts and Enterobacteriaceae counts of chicken meat in Ibadan metropolis area.**

No significant difference was observed when the mean aerobic plate count and mean enterobacteriaceae counts in log_10_ cfu/g of chicken from one market was compared with one another at p < 0.05 with 5% level of error. The level of bacterial contamination from one local government area to another showed only slight differences as reflected in the bar charts drawn in Figure 1. Mean APC was found to be higher than ENT counts interpreted as higher rates of aerobic bacteria contaminants on chicken.

#### Aerobic plate count and Enterobacteriaceae counts in turkey

Using ANOVA and multiple comparisons, no significant differences was observed when the mean total aerobic plate counts of turkey were calculated individually for each LGA and when they were compared one to another. Mean (cfu/g) ± SD aerobic plate count (APC) for all the LGA’s was calculated as 7.42 ± 1.00. No significant differences was observed when the mean Enterobacteriaceae counts of turkey were calculated individually for each LGA and when they were compared one to another using ANOVA and multiple comparisons. Mean (cfu/g) ± SD Enterobacteriaceae counts for all the LGA’s was calculated as 6.74 ± 2.00. Table 2 shows differences obtained amongst LGA’s.

**Table 2:** **Aerobic plate counts and enterobacteriaceae counts for turkey meat in Ibadan metropolis**

Ibadan LGA	Sample size	Aerobic plate count cfu/g	Enterobacteriaceae count cfu/g
	n	Mean ± SD	Mean ± SD
Ibadan North	18	7.16 ± 1.40	6.87 ± 2.00
Ibadan North-West	5	7.55 ± 0.49	7.18 ± 0.38
Ibadan North-East	5	7.53 ± 0.54	5.88 ± 2.30
Ibadan South- West	11	7.68 ± 0.46	6.63 ± 2.57
Ibadan South-East	7	7.51 ± 0.86	6.90 ± 1.77
Total	46	7.42 ± 1.00	6.74 ± 2.00

key : LGA = Local Government Area, n = sample numbers collected, SD = standard deviation, cfu = colony forming unit, g = gram.

**Figure 2:**
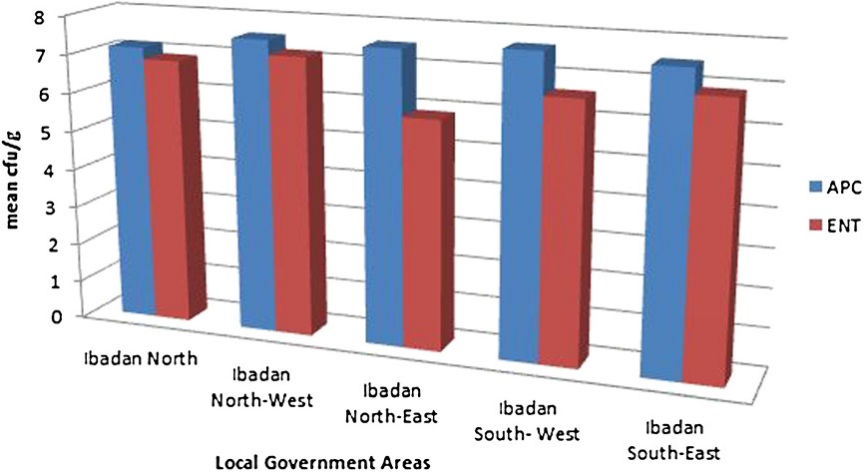
**Aerobic plate counts and Enterobacteriaceae counts of turkey meat in Ibadan metropolis area.**

No significant difference was observed when the mean aerobic plate count and mean enterobacteriaceae counts in log_10_ cfu/g of turkey from one market was compared to other market at p < 0.05 with only slight differences illustrated in their means as shown in the bar charts. APC in turkey is however higher than that obtained from Enterobacteriaceae counts, meaning that a higher contamination by aerobic bacteria was seen in turkey than enteric bacteria. Differences are illustrated in charts as seen in Figure 2.

**Figure 3:**
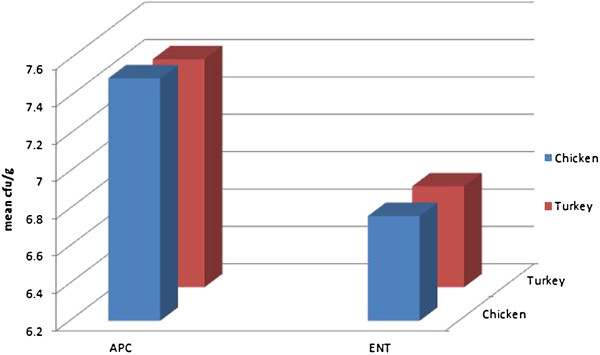
**Distribution between Aerobic Plate Counts (APC) and Enterobacteriaceae counts (ENT) in poultry from retail market.**

**Figure 4:**
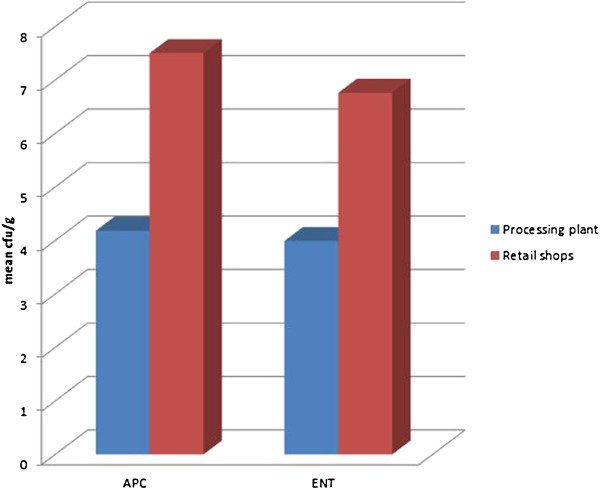
**Aerobic plate counts and enterobacteriaceae counts in chicken from retail markets and processing plant.**

Figure 3 shows APC and ENT distribution obtained from retail poultry meat. Table 3 shows a representation and comparism between Aerobic plate count and Enterobacteriaceae counts obtained in chicken from retail market and processing plant which is graphically represented in Figure 4.

**Table 3:** **Aerobic plate counts and enterobacteriaceae counts in chicken meat from processing plant in comparison to those from retail market**

Ibadan metropolis	Sample size	Aerobic plate count cfu/g	Enterobacteriaceae count cfu/g
	n	Mean ± SD	Mean ± SD
Processing plant	53	4.18 ± 1.47	3.99 ± 2.53
Retail shops	53	7.50 ± 0.94	6.76 ± 1.93
Total	106	5.84 ± 2.07	5.38 ± 2.64

n = sample numbers collected, SD = standard deviation, cfu = colony forming unit, g = gram.

### Extent of *Salmonella*and *Escherichia coli*contamination

**Figure 5:**
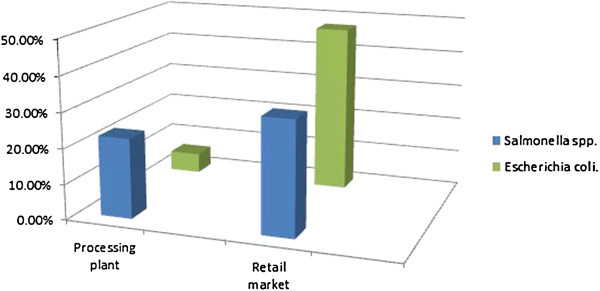
***Salmonella spp.*** **and** ***Escherichia coli*** **in chicken from Processing plant and Retail market.**

From the one-hundred and six (106) chicken sampled comprising equal numbers of fifty-three from both retail market and processing plant, a total of twenty-nine (29) *Salmonella* spp. isolates, seventeen (17) from retail markets and twelve (12) from the processing plant were obtained while for *Escherichia coli* a total of twenty-eight (28) comprising twenty-five (25) from the retail market and three (3) from the processing plant were obtained. Percentages of isolates are shown in Figure 5.

#### Retail market

From the ninety-nine (99) poultry meat sampled (53 chicken and 46 turkey), thirty-three (33) *Salmonella* isolates (17 from chicken and 16 from turkey) were obtained at prevalence of 33% while a total of forty-three (43) *Escherichia coli* isolates (25 from chicken and 18 from turkey) were obtained at a prevalence of 43.4%.

#### Processing plant

From the 53 chicken samples obtained from the processing plant, twelve (12) *Salmonella* spp. isolates were obtained with prevalence of 22.6% while three (3) *Escherichia coli* isolates were obtained with prevalence of 5.7%.

Antibiotic sensitivity test carried out on both *Salmonella* and *Escherichia coli* isolates shows the pattern of sensitivity represented in Tables 4 and 5 respectively.

**Table 4:** **Antimicrobial susceptibility pattern for** ***Salmonella enterica*** **spp. (n = 45)**

Antibiotics (μg)	Totally sensitive		Intermediate		Resistant	
	No.		No.		No.	
**Aug (30)**	0	0%	0	0%	45	100%
**Ofl (5)**	33	73%	5	11%	7	16%
**Gen (10)**	23	51%	9	20%	13	29%
**Nal (30)**	0	0%	12	27%	33	73%
**Nit (200)**	10	22%	10	22%	25	56%
**Cot (25)**	3	7%	0	0%	42	93%
**Tet (25)**	0	0%	3	5%	42	93%

Key: No. - numbers of isolates, Aug (Augmentin), Ofl (Ofloxacin),Gen (Gentamicin), Nal (Nalidixic acid), Nit (Nitrofurantoin), Cot (Cotrimaxazole) and Tet (Tetracycline).

**Table 5:** **Antimicrobial susceptibility pattern for** ***Escherichia coli*** **(n = 46)**

Antibiotics (μg)	Sensitive		Intermediate		Resistance	
	No.		No.		No.	
**Aug (30)**	0	0%	0	0%	46	100%
**Ofl (5)**	29	63%	6	13%	11	24%
**Gen (10)**	14	30.4%	19	41.3%	13	28.3%
**Nal (30)**	5	11%	14	30%	27	59%
**Nit (200)**	17	37%	5	11%	24	52%
**Cot (25)**	3	7%	2	4%	41	89%
**Tet (25)**	8	17%	0	0%	38	83%

Key: n - numbers of isolates, Aug (Augmentin), Ofl (Ofloxacin), Gen (Gentamicin), Nal (Nalidixic acid), Nit (Nitrofurantoin), Cot (Cotrimaxazole) and Tet (Tetracycline).

## Discussion

### Aerobic plate count and Enterobacteriaceae counts of frozen poultry

#### Retail market

ENT counts were observed to be higher than APC for both chicken and turkey meat. This is because the agar used in determining APC is sensitive to all aerobic bacteria thereby allowing a wide range of bacteria to grow. MCA used in determining ENT is however limited in the range of bacteria that it allows its growth as it is only selective for enteric bacteria such as *Escherichia*, *Shigella*, *Salmonella*, *Edwardsiella*, *Citrobacter*, *Yersinia*, *Klebsiella*, *Enterobacter*, *Serratia*, *Proteus*, *Morganella* and *Providencia* as noted by Warren (2007). Slight differences were observed when comparing chicken with turkey meat. It however goes to say the samples from which these growths were obtained are contaminated at levels exceeding limits by food regulatory bodies, a reflection of the condition of the whole carcass meant for human consumption.

#### Processing plant

Mean aerobic plate count was calculated as 4.18 cfu/g which is less than 6.7 cfu/g obtained from a processing plant in Morocco (Cohen et al. 2007). Significant difference was observed in aerobic plate counts and enterobacteriaceae counts of chicken between processing plant and retail market at 0.006. Mean cfu/g ± SD of 3.99 ± 2.53 was obtained from processing plant. Although there were differences in Aerobic plate count and Enterobacteriaceae counts obtained between processing plant and retail market, where APC were noted to be higher than ENT counts, counts are reflection of the general sanitary condition of the carcasses and basically at high levels than permitted on such food.

### *Salmonella*and *Escherichia coli*

#### Retail markets

*Salmonella* in more than 25 g of poultry meat is considered unsafe for human consumption. From the work understudied, *Salmonella* at levels higher than the recommended limits was obtained from both chicken and turkey. 33.3% (33) of poultry meat (chicken and turkey) from the ninety-nine samples obtained were contaminated with *Salmonella*. This percentage is higher than the 11.1% obtained in Calabar metropolis (Ukut et al. 2010) and the 2% from Osogbo (Adesiji et al. 2011) all from Nigeria. However, levels are indicative of the fact that if proper cooking is not done, *Salmonella* food-borne infection is most likely to occur.

*E. coli* is recommended to be totally absent from poultry meat before such can be considered fit for human consumption. Levels obtained from work are reflections of the high rates obtained from markets. 43.4% (43/99) obtained from work is very high in comparison to 11.1% and 16% from Osogbo (Adesiji et al. 2011) and Calabar metropolis (Ukut et al. 2010) respectively. 11.3% prevalence reported from neighboring Cameron (Nzouankeu et al. 2010) is however lower than that from Nigeria even though we both have the same source of imports of these poultry meat. The rate of *E. coli* obtained is indicative that poultry meats obtained from sourced areas were unfit for human consumption in accordance with criterion of recommended limits by foreign food agencies. Poultry meat obtained from these markets should therefore be properly cooked to denature toxin produced by the organism as well as the organism such that consumption will not pose health-risks to human population.

#### Processing plant

The processing plant had a prevalence rate of 22.6% from chicken meat when compared to 1.56% from a processing plant in Morocco (Cohen et al. 2007) and 20% in USA processing plant (Russell 2009). Albeit, *Salmonella* contamination in poultry products from the processing plant is primarily due to cross contamination by physical contact during carcass processing such as improper cleaning and disinfection of processing lines, improper chilling and storage temperature, poor worker hygiene and infestation with rodents and insects.

The prevalence of *Escherichia coli* up to 94% was detected in chicken meat samples in the Netherlands which is quite alarming (HCV New Drug Research 2011) when compared to 5.7% obtained from study. These poultry products get contaminated with *Escherichia coli* along processing line when intestinal contents accidentally spills on the meat been processed or due to contaminated equipments/storage/transporting facilities.

### Antibiotic sensitivity tests

#### *Salmonella*antibiotic sensitivity

The development of quinolone-resistant *Salmonella* resistant strains, especially those of poultry meat origin (Miranda et al. 2009), is gradually leading to an epidermic (White et al. 2001; Velge et al. 2005). Nalidixic acid is able to develop resistance quite rapidly. Several studies have also shown that resistance to nalidixic acid and decreased susceptibility to Fluoroquinolones have increased among *Salmonella* spp from food animals (Aarestrup et al. 2003; The Merck Manual 1998, eight edition). Table 4 shows *Salmonella* resistance pattern of ofloxacin at 16% is a reflection that it is least resistant to the organism, whereas the Fluoroquinolones generally have been previously known to have a good sensitivity to *Salmonella* (The Merck Manual 1998, eight edition). 100% resistance of *Salmonella* from poultry sources to augmentin is in line with previous works (Ezekiel et al. 2011) even though augmentin has been known to be of variable resistance. Tetracycline have repeatedly shown high levels of resistance of 90%–100% (Sakaridis et al. 2011) to which the work carried out was in agreement.

#### *E. coli*antibiotic sensitivity

Resistance patterns to tetracycline and gentamicin were high compared to previous works which showed lower resistance (Musgrove et al. 2006). Resistance of 59% Nalidixic acid and 28.3% gentamicin obtained from research is almost similar to 70.3% Nalidixic acid and 24.3% gentamicin observed in isolates of poultry meat in Saudi Arabia (Altalhi et al. 2010). Augmentin has variable resistance to the *E. coli* but work showed 100% resistance and Ofloxacin which is known to have a good sensitivity to *E. coli*, showed the lowest resistance in work. From documented research, *E. coli* resistance to ciprofloxacin has been found to be higher in broilers than the microorganisms isolated from other sources such as pigs. This might be due to the widespread use of these antimicrobial agents (Miranda et al. 2008).

The fluoroquinolones are active against a wide range of bacteria including enteric bacteria but are gradually lossing this ability (Aarestrup et al. 2003) hence of increasing public health concern. Of necessity is the Veterinary therapeutic use of antimcrobials. However, because treated chickens are sent to the slaughter house immediately after the withdrawal period of antimicrobial agents used, resistant bacteria could be a risk to public health after the slaughter and processing of such birds. It therefore seems necessary to extend the withdrawal period after antimicrobial therapy (Miranda et al. 2008).

## Conclusion

1. This study has revealed that imported poultry meat in Ibadan markets are contaminated with *Salmonella* and *E. coli* at levels higher than those obtained locally from a processing plant.
2. *Salmonella* and *E. coli* are gradually gaining more resistant to antibiotics.

### Recommendation

1. Standards of food quality as regards imports of meat and meat-products should be setup and demand for quality certification of products shown before the products are allowed into the country. These standards should have a legal backing and should stand for all imports of raw and ready-to-eat foods.
2. It is important that the use of the HACCP (Hazard Analysis Critical Control point) approach, based on the use of multi-functional strategies (combining the innovative use of sanitizers and modern disinfection techniques) and supervised by professional food handlers and food regulators with a visionary commitment by management from the production, through the processing, preservation, handling and final preparatory stages, be imposed to help eliminate or reduce significantly the prevalence of *Salmonella*, *Escherichia coli* and other food-borne pathogens/contaminants and the consequent food poisoning in the society.
3. The indiscriminate use of antibiotics should be cautioned because antibiotics will soon completely loose their effectiveness against microorganisms especially as *Escherichia coli* acquires antimicrobial resistance faster than other conventional bacteria due to increase resistance.
4. Washing of hands properly before sales of meat or usage of proper clothing such as hand gloves, nose masks and head covers.
5. Proper cold-preservation of meat will prevent multiplication of contaminants.
6. Cooking at high temperatures of 100°C will help to eliminate pathogens before consumption.
